# Application of Artificial Intelligence and Sensor Fusion for Soil Organic Matter Prediction

**DOI:** 10.3390/s24072357

**Published:** 2024-04-08

**Authors:** Md Jasim Uddin, Jordan Sherrell, Anahita Emami, Meysam Khaleghian

**Affiliations:** College of Science and Engineering, Texas State University, San Marcos, TX 78666, USA; muddin2@ncsu.edu (M.J.U.); jms621@txstate.edu (J.S.)

**Keywords:** Artificial Intelligence, sensor fusion, soil organic matter

## Abstract

Soil organic matter (SOM) is one of the best indicators to assess soil health and understand soil productivity and fertility. Therefore, measuring SOM content is a fundamental practice in soil science and agricultural research. The traditional approach (oven-dry) of measuring SOM is a costly, arduous, and time-consuming process. However, the integration of cutting-edge technology can significantly aid in the prediction of SOM, presenting a promising alternative to traditional methods. In this study, we tested the hypothesis that an accurate estimate of SOM might be obtained by combining the ground-based sensor-captured soil parameters and soil analysis data along with drone images of the farm. The data are gathered using three different methods: ground-based sensors detect soil parameters such as temperature, pH, humidity, nitrogen, phosphorous, and potassium of the soil; aerial photos taken by UAVs display the vegetative index (NDVI); and the Haney test of soil analysis reports measured in a lab from collected samples. Our datasets combined the soil parameters collected using ground-based sensors, soil analysis reports, and NDVI content of farms to perform the data analysis to predict SOM using different machine learning algorithms. We incorporated regression and ANOVA for analyzing the dataset and explored seven different machine learning algorithms, such as linear regression, Ridge regression, Lasso regression, random forest regression, Elastic Net regression, support vector machine, and Stochastic Gradient Descent regression to predict the soil organic matter content using other parameters as predictors.

## 1. Introduction

The agriculture industry is undergoing a tremendous transformation by embracing technology to increase crop output and enhance decision making when analyzing soil properties. It has become vital to adapt to cutting-edge technology, including robotics for weeding, picking, crop segregation, harvesting, and packing, cloud-based environmental monitoring, remote sensing IoT-based agriculture, and autonomous robotic monitoring and management systems.

According to studies conducted by Bauer and Black [1], soil organic matter (SOM) has a significant role in enhancing crop health, which leads to an increase in crop growth. To assess soil fertility, it is essential for rapid and accurate measurement of soil organic matter (SOM), organic carbon, and total nitrogen (TN). It is believed that soil fertility, porosity, and nutrient supply decrease due to the reduction in SOM [2,3]. SOM contains about 58% carbon, making the soil organic carbon (SOC) one of the major indicators of SOM content in soil [4]. Lal [5] suggested that the critical or threshold level of SOC in temperate zone soils is approximately 2%, while in tropical soils, it is around 1%. Schjønning et al. [6] observed that the presence of soil organic matter (SOM) had a beneficial impact on crop yield, as it reduced the amount of mineral nitrogen (N) required to achieve the maximum crop yield. The author further concluded that SOM contributes to crop production beyond its role in providing nutrients. SOM primarily affects soil tilth, rooting depth, nitrogen release, and infiltration and retention of soil. Therefore, in order to comprehend the necessary inputs, crop farmers must have accurate information about SOM.

In addition, SOM serves as a reservoir for essential nutrients, including nitrogen, phosphorus, and sulfur, which are vital for plant growth and microbial activity and enhance soil structure, promoting water retention and drainage, thus mitigating the risks of erosion and runoff [7]. Moreover, SOM contributes to soil carbon sequestration [8,9], playing a crucial role in mitigating climate change by reducing atmospheric carbon dioxide levels. Furthermore, SOM promotes microbial diversity and activity, which are essential for nutrient cycling, disease suppression, and the degradation of pollutants in soil activity [10,11,12,13,14,15,16]. Thus, SOM plays an important role in maintaining soil and environmental health through various mechanisms.

The conventional approach (oven-dry method) of calculating the SOM is expensive and time-consuming [17], primarily due to the expensive and time-intensive nature of soil sampling procedures [18]. Electromagnetic induction sensors, along with topographic parameters, are being used to estimate the soil organic matter by interpolation methods [19]. Techniques like inverse distance weighting, geostatistical, ordinary kriging (OK), cokriging (COK), and regression kriging either with linear models (LM-RK) or with random forest (RF-RK) were also used to obtain SOM distribution maps. In precision agriculture, several studies have used ground-in sensors and wireless network sensors to predict soil characteristics [20,21,22,23]. Coelho et al. [22] created a method for data collecting parameters aimed toward an automated irrigation system. Kweon and Maxton [21] created an affordable, portable optical sensor (spectrometer) for assessing SOM to assist farmers in quickly making informed decisions using field data that are currently being collected.

Unmanned aerial vehicles (UAVs) have become one of the popular data collection tools in practically every business [24,25,26,27]. Compared to other techniques of data collecting, UAVs are said to be quick and effective. High-resolution satellite imagery has a cost and availability limit for use in precision agriculture (PA). UAVs have been shown to be accessible and affordable remote sensing equipment [28]. Data collection, field variability mapping, decision making, and management are all steps of PA procedures that are included in remote sensing [29,30]. Unmanned aerial systems (UASs) are more advantageous in studying the use of high-resolution photographs in PA since satellite photography has spatial resolutions compared to UASs. Many studies and reports have focused on soil reflectance as an important method of estimating soil organic matter (SOM) [31,32,33,34]. It is possible to determine parameters such as surface soil properties [35], water stress [36], vegetation cover [37], nitrogen content [38,39,40,41], crop height [42], above-ground biomass [38], crop yield [43], weed extent [44], and crop species [45,46] using vegetative indices such as the Normalized Difference Vegetation Index (NDVI), Normalized Difference Red Edge Index (NDRE), Soil Adjusted Vegetation Index (SAVI), and Green NDVI.

Near-infrared (NIR) spectroscopy is a rapid and relatively inexpensive technique with minimal sample preparation and no hazardous chemicals that can be used to measure several soil properties from a single scan. Therefore, several studies [47,48,49,50] have explored the efficacy of near-infrared (NIR) spectroscopy in detecting SOM content across various soil types and ecosystems. The authors of [51] found that the soil organic carbon predictions using NIR were most inaccurate for soils with a high sand content. The authors of [52] proposed a statistical approach to improve the prediction of SOM using NIR. The authors of [53] investigated the efficiency of NIR for evaluating SOM in saline–alkali soil. The authors of [54] showed that using deep learning methods allows better prediction of the SOM content from NIR. Hummel et al. [55] created a portable spectrophotometer to calculate the SOM over a large agricultural area and demonstrated that there was a strong correlation between the reflectance of NIR spectral areas and the SOM. Stiglitz et al. [56] suggested an inexpensive color sensor for the rapid assessment of soil organic carbon and total nitrogen. Ge et al. [57] analyzed hyperspectral vegetation data obtained from unmanned aerial vehicles (UAVs) to calculate the soil moisture content. Zheng et al. [58] proposed an innovative technique to survey coconut trees by incorporating NDVI indices using satellite imageries. The authors incorporated three modules to solve the problem of detecting small objects by distinguishing the features and comparing them with a predefined context semantic dataset.

Eskandari et al. [59] used machine learning (ML) and statistical models to meta-analyze the unmanned aerial vehicles (UAVs) photography application. Jay et al. [60] suggested using multispectral data from UAVs to obtain canopy variables and looked into how centimeter-scale photography can help with leaf and canopy variable estimation. Heil et al. [61] implemented UAVs and ML to estimate the fine mapping of SOM in sugar beets. The authors used UAVs to capture low-altitude high-resolution images of a crop field to generate a dataset of color and topographic covariates of crops for the models using Pix4Dmapper, and SOM was calculated using the loss-on-ignition (LOI) method, oven-drying. They utilized partial least square regression (PLSR), the ensemble algorithm random forest (RF), and artificial neural networks (ANNs) to map the SOM of the field, and ten-fold cross-validation was used to evaluate the point support forecasts. According to their finding, RF provided the best estimation of SOM with an RMSE of 0.13 and R^2^ of 0.68. Partel et al. [62] developed an automated crop sprayer by using sensor fusion and machine vision. The authors used LiDaR to identify tree height, cameras for imageries, and GPS for locating and navigating through the tree, and they incorporated a controller unit to communicate through machine vision to spray crops wherever necessary. Sothe et al. [63] attempted to compute the spatial and vertical distributions of SOC concentration using a three-dimensional (3D) machine learning approach and 40 spatial predictors collected from 20 years of optical and microwave satellite measurements. In a 10-times repeated five-fold cross-validation approach, an RF model with 25 variables produced the best results, predicting the country’s SOC with an RMSE of 0.58 and an R^2^ of 0.83.

The main objective of this study is to integrate ground-based sensors and drone technology, leveraging machine learning techniques to predict SOM content with greater accuracy compared to prior studies. Our proposed strategy aims to provide farmers with a SOM predictive algorithm to enhance soil management practices and support sustainable agriculture. To facilitate the analysis, the ground-in sensors capture the SOM-affecting variables such as soil temperature, relative humidity, pH, nitrogen, phosphorus, and potassium content. A multispectral drone will be used to capture field images, while soil analysis data will be obtained from laboratory results. Pix4Dfields software version 1.10.1 will be used to process images taken by the drone to determine NDVI. Machine learning algorithms will be incorporated to calculate SOM using all collected soil parameters and NDVI data.

## 2. Methodology

### 2.1. Research Design

**Figure 1:**
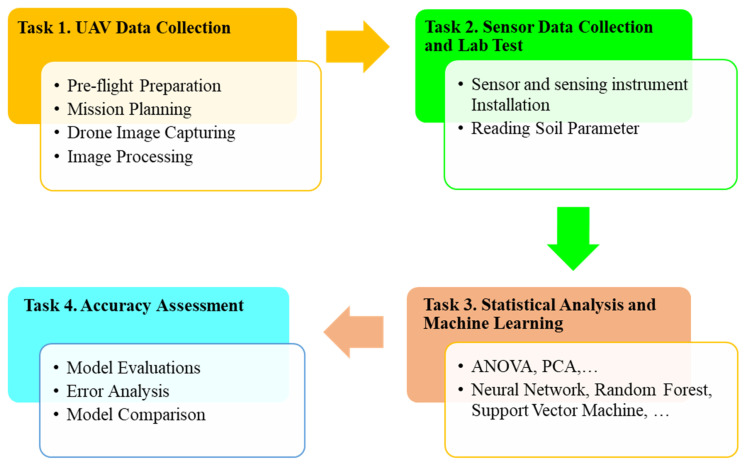
Project workflow.

Figure 1 shows the typical data processing workflow of predicting soil organic matter content using soil sensor data, UAV images, and soil analysis reports. To conduct this study, a hybrid methodology combining ground-based soil sensors and the DJI Multispectral P4 UAV was used. The Multispectral UAV was initially flown over the chosen farm to collect crop image data all over the property. The poor/weak zone of the crop was determined by collecting NDVI and NDRE data after post-processing the acquired photos with an image processing program such as Pix4Dfields. To estimate the SOM present in the soil, the second step entails installing a set of soil sensors in the zone that was identified as being weak and measuring the approximations for crop environmental factors like temperature, moisture, texture, salinity and acidity, vegetation, and biomass production. A significant amount of sample data was gathered from UAVs and installed ground-based sensors, and the soil analysis data and the dataset were processed before being incorporated into a machine learning algorithm. The collected data from ground-in sensors, as well as NDVI values of those zones, was incorporated in machine learning algorithms to determine the SOM more accurately, and based on the predicted accuracy level, we were able to define which machine learning algorithms will be suitable to predict the SOM level of the soil.

Figure 1 shows the project workflow. For our research, we did not use the methods of calculation of NDVI provided by the P4 multispectral drone. We used Pix4Dfields software to measure the NDVI by uploading the drone images and cascading the images to generate a complete image of the selected area. Then, we used the NDVI option to generate a histogram of different NDVI based on the reflectance of light from the crop. The software provided a range of NDVI values for the land and also showed different zones with different NDVI values.

As a part of our data collection of soil parameters, we used ground-in sensors to measure soil pH, temperature, humidity, nitrogen, phosphorous, potassium, etc. We installed sensors in different zones with distinct NDVI values. All the above-mentioned parameters were measured using our selected sensor. Soil samples were collected from those zones and sent for soil analysis.

### 2.2. Research Sites

**Figure 2:**
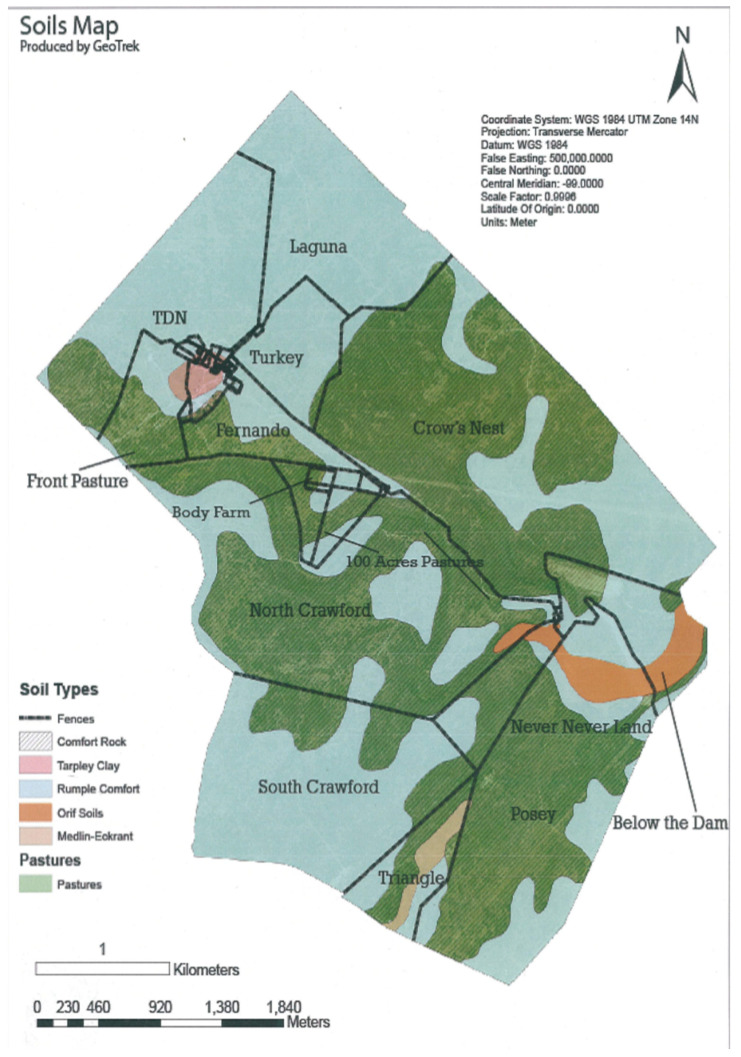
Soil map of Freeman Ranch (source: TXstate).

The research study was conducted in three different locations, each with distinct soil types. The first study area was Freeman Ranch, located in San Marcos. For this research, we collected soil samples from four different regions within Freeman Ranch, each representing a different soil type. The soil map of Freeman is illustrated in Figure 2. The second study area was TXstate Star Park, another research facility owned by Texas State University. We conducted a field survey at TXstate Star Park in March 2023 and collected soil data between March and May 2023. We collected our data from one soil type of Star Park farm. Our third research location was the Montesino Ranch, located in Wimberly, Texas. The Montesino Ranch is a privately owned ranch with different farm areas with different soil types based on their crops. They maintain the soil types in different farm areas using cow grazing, horse grazing, and fertilizer, as well as maintaining well-measured soil parameters based on their farming demand. We collected soil samples from seven farm areas with different soil types.

**Figure 3:**
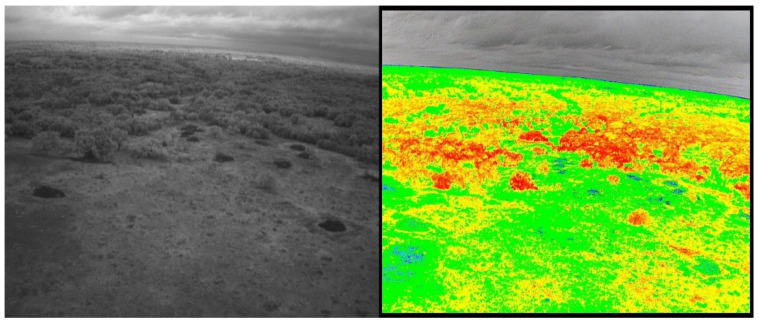
Freeman Ranch: narrow-band image (**left**), multispectral image (**right**).

**Figure 4:**
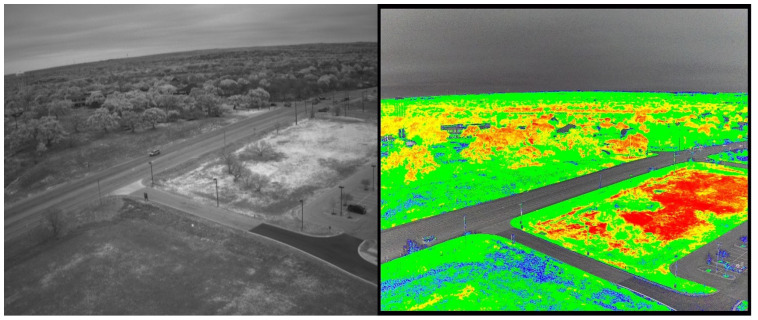
TXstate Star Park: narrow-band image (**left**), multispectral image (**right**).

**Figure 5:**
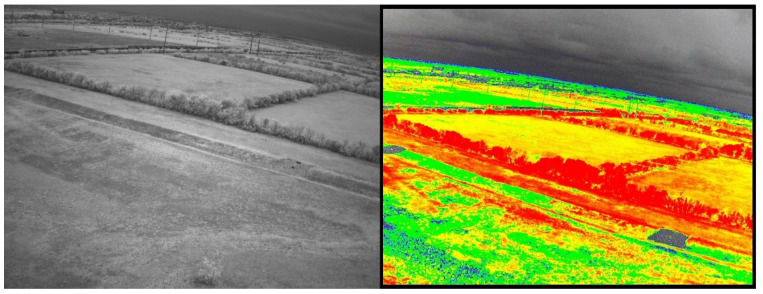
Montesino Ranch: narrow-band image (**left**), multispectral image (**right**).

We surveyed the Freeman Ranch field using UAV and analyzed the images for NDVI in the 1st week of August 2022 and completed the soil parameters and soil sample of 100 data in August 2022, September 2022, and March 2023. In the TXstate Star Park, we surveyed the field in October 2022 and March 2023 and completed the soil data collection in March–May 2023. We collected 200 samples from the TXstate Star Park from one soil type. In Montesino Ranch, we completed a UAV survey on May 7 and collected data in May 2023. We measured soil parameters from seven farms, each with different soil types, and collected a total of 200 soil samples from the Montesino Ranch. The multispectral and narrow-band images of Freeman Ranch, TXstate Star Park, and the Montesino Ranch are illustrated in Figure 3, Figure 4, and Figure 5, respectively.

### 2.3. Sensor Installation and Soil Parameter Measurement

Different sensor installation zones were selected based on the NDVI values found after the image processing was performed. We dug holes in selected zones for sensor installation using an Earth Auger Drill. The depth of the hole was >1 ft, and the diameter of the hole was 8 inches. The sensors were installed vertically in the direction of the sensor probe. Sensors were installed at least 3 ft apart from each other so that we could have two distinct datasets of soil parameters. During the hole digging, we tried to minimize the disruption to the surrounding soil and prevent the mixing of soil layers by controlling the drilling process and avoiding excessive disturbance to the soil structure. Moreover, we installed the sensors gently in vertical directions at the bottom of the hole to record the parameters as closely as possible to the undisturbed soil layer to enhance the accuracy of our measurements.

After installing the sensor, we covered the hole tightly with soil and connected the battery and laptop via RS485. The sensor displays the soil parameters based on the command supplied through sensor software. In the sensor interface, there are options to select different soil parameters based on the requirement, and after selecting the desired parameter, sensors need to be connected. Through the sensor interface, the software sends a command to read data of the selected parameter, and the sensor sends the parameter data to the laptop to be displayed.

We collected 100 datasets of soil parameters by installing a sensor 1 ft deep in the soil on different soil types in Freeman Ranch. We collected soil samples from every 100 zones in zip-lock bags to send those samples to a soil lab to measure SOM content. We have also collected 200 datasets of soil parameters from TXstate Star Park Field and collected soil samples in a zip-lock bag as well. Finally, we measured 200 datasets of soil parameters from the Montesino Ranch and collected soil samples from each zone in a zip-lock bag. All 500 soil samples, which were collected from different zones of these fields, were sent to Regen Ag Lab, Nebraska, to analyze and measure the Haney test and SOM content for each soil sample.

### 2.4. Data Analysis

We collected 500 datasets of soil parameters from the selected research farms. We used soil-integrated sensors to measure soil temperature, soil humidity, soil pH, nitrogen, phosphorous, and potassium and calculated the NDVI values of the farm by processing multispectral images collected by drone survey. Additionally, we collected measurement data from Haney tests performed on collected samples, collaborating with a specialized soil analysis laboratory based in Nebraska. NDVI and soil parameters were considered as our input variables, while SOM content served as the output for training and testing our predictive models. After getting all the necessary parameters, the design of experiments (DOE) processes, such as Analysis of Variance (ANOVA), regression, etc., were implemented to determine the variability of the dataset. Initial statistical data were analyzed using OriginPro. After SOM was evaluated and analyzed, machine learning algorithms such as linear regression, Ridge regression, Elastic Net regression, Lasso regression, random forest, Stochastic Gradient Descent regression, and support vector machine regression were implemented to predict the SOM. Then, the prediction accuracy as per each ML algorithm was summarized.

## 3. Results

### 3.1. UAV Survey

**Figure 6:**
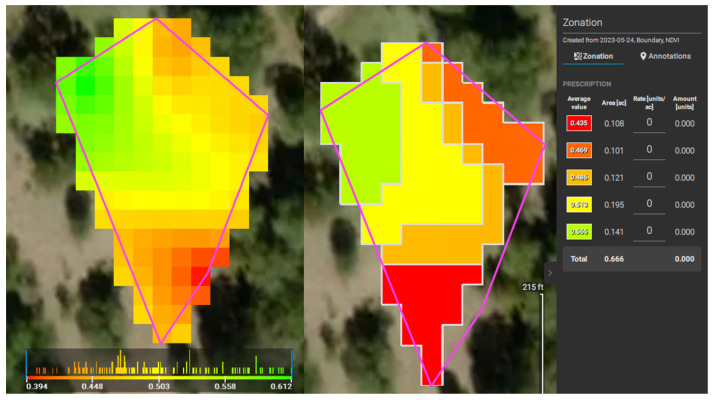
NDVIs of Freeman Ranch.

**Figure 7:**
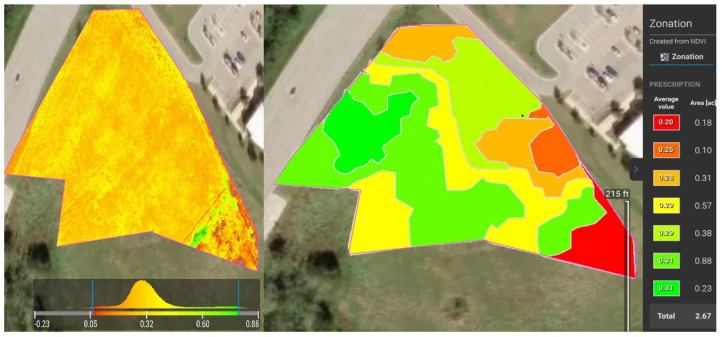
NDVIs of TXstate Star Park Farm in Green-Red Mode (**left**) and Zonation (**right**).

**Figure 8:**
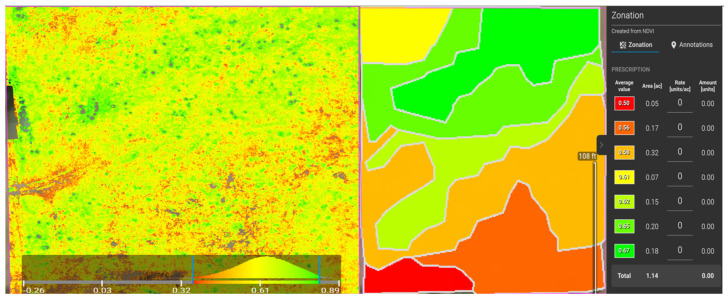
NDVIs of the Montesino Ranch Farm in Green-Red Mode (**left**) and Zonation (**right**).

The UAV surveys on Freeman Ranch, TXstate Star Park, and Montesino Ranch were conducted on 2 August 2022, 7 February 2023, and 11 May 2023, respectively, and each survey was conducted on a cloudless, sunny day. On the Star Park field, before installing the sensor, we designed a flight path for the drone mission to capture images of the field. In the DJI P4 Multispectral drone, there are two modes of image capture: RGB and NDVI. In RGB mode, the drone captures one RGB image and five narrow-band photos, whereas in multispectral mode, it captures one multispectral image and five narrow-band images for every image in the mission. After the mission was completed, the images were processed using Pix4Dfields image processing software to determine the zones on the field based on the NDVI values. Pix4Dfields image processing software provides a histogram plot of NDVI after processing the images. The Pix4Dfields software has an advanced option to convert the NDVI image into 2–7 zones based on the NDVI values. The software interface shows the average value of NDVI in those zones. We dug up soil using an auger in those zones and installed sensors inside those holes to collect the soil parameters such as temperature, soil pH, nitrogen, phosphorous, and potassium. The process flow of proprietary Pix4Dfields can be explained as camera positions and angles are approximated, geometric photo adjustment is applied, and data points made up of matched points from overlapping images are generated. The multispectral images with NDVI values in Green-Red Mode and Zonation of the NDVI images of Freeman Ranch, TXstate Star Park, and the Montesino Ranch are illustrated in Figure 6, Figure 7, and Figure 8, respectively.

### 3.2. Results of Data Analysis

#### 3.2.1. Regression Analysis

**Figure 9:**
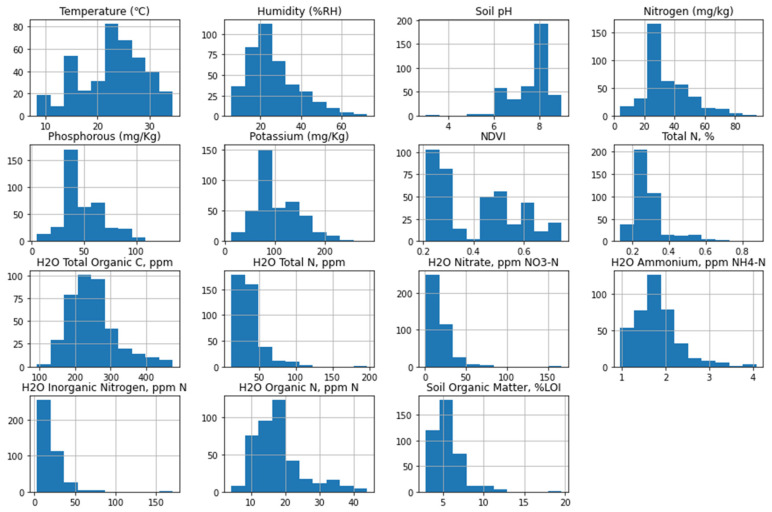
Histogram plot distribution of soil parameters.

The histogram plots of soil parameters are depicted in Figure 9. We can see that temperature and pH distribution is left-skewed distribution, which means that the mean values of these two parameters are less than their medians. On the other hand, the distribution of nitrogen, phosphorous, and potassium are right-skewed distribution, resulting in higher means than their medians. Similarly, all the other parameter’s distributions are also right-skewed distributions, and hence, the means are higher than the medians.

Table 1 presents the descriptive statistics of soil parameters. Throughout the sample period, the average temperature was 22.81 °C, with a standard deviation of 6.13 °C. The lowest recorded temperature was 8.30 °C, while 34.50 °C was the highest. These numbers point to a moderate degree of temperature fluctuation among the research sites. With a standard deviation of 12.20%RH, the mean humidity level was 26.37%RH. The minimum and maximum humidity readings were 5.60%RH and 72.70%RH, respectively. These numbers show a wide range in humidity levels, which reflects variations in moisture content between the research regions. The average soil pH was 7.63, with a 0.92 standard deviation. The median pH was 8.03, and the range of pH values was 3.0 to 9.00. This shows a soil pH range of slightly acidic to slightly alkaline, with moderate pH level fluctuation. The statistical analysis shows that the key soil nutrient concentrations varied greatly in the soil. Mean nitrogen, phosphorous, and potassium (NPK) levels were 34.99 mg/kg, 49.35 mg/kg, and 107.14 mg/kg, with a standard deviation of 15.21 mg/kg, 19.70 mg/kg, and 42.86 mg/kg, respectively, whereas the range of NPK level was 4–94 mg/kg, 5–135 mg/kg, and 13–285 mg/kg, respectively. The quantities of nitrogen, phosphorus, and potassium in these different types of soils vary significantly. The mean NDVI was 0.41, with a standard deviation of 0.16. Similarly, the Haney test result shows that there are significant variances in terms of Haney content in these farmlands. Finally, the soil organic matter content varied from 2.9% to 19.6%, with a mean of 5.56% and a standard deviation of 1.75%. The statistical analysis of the collected soil parameters provides significant insights into the relationship between independent and dependent variables. The regression equation is expressed as follows.

Regression Equation:
Soil Organic Matter, %LOI = 0.254 + 0.0471 Temperature (°C) − 0.00296 Humidity (%RH)
− 0.0818 Soil pH + 0.0771 Nitrogen (mg/kg) 
− 0.0749 Phosphorous (mg/kg) + 0.01457 Potassium (mg/kg) 
− 0.926 NDVI + 16.387 Total N (%)


The regression equation shows that temperature, nitrogen, phosphorous, potassium, total N, and total organic C have a positive correlation with soil organic matter. On the other hand, humidity, soil pH, NDVI, H_2_O total N, and H_2_O organic N have a negative correlation with soil organic matter. Conant et al. [64] and Kirschbaum [65] confirmed that high temperature increases the decomposition of SOM. Studies show that SOM has a positive correlation with humidity [66], nitrogen and total N [67], phosphorous [68], and potassium [69] and a negative correlation with temperature (Conant et al. [64] and Kirschbaum [65]), soil pH [70], and NDVI [71].

From Table 2, the *p*-value of soil pH and humidity is much higher than the level of significance (0.005), which indicates that soil pH and humidity are not influential parameters for predicting soil organic matter. The *p*-value of NDVI is almost similar to the significance threshold, suggesting strong relationships with soil organic matter. On the other hand, temperature, NPK, total N, H_2_O total N, H_2_O total organic C, and H_2_O total organic N have a strong correlation with the SOM.

As presented in Table 3, the regression model has an R-squared value of 0.7682, which indicates that the model can explain 76.82% of total variability for predicting soil organic matter.

These findings underscore the multifaceted nature of SOM dynamics and emphasize the potential of NDVI and select soil parameters as predictors of SOM content. In our study, soil pH and humidity exhibit limited correlation with SOM, NDVI, NPK levels, and total nitrogen, and water-soluble organic components display strong correlations with SOM.

#### 3.2.2. ANOVA Analysis

The results of the ANOVA analysis are presented in Table 4. The ANOVA analysis shows that soil pH and humidity have a weak relationship with soil organic matter in our case studies. This finding is in line with the previous studies in which Hong et al. [72] demonstrated that soil rich in SOM leads to lower pH, and Qu et al. [73] identified that soil moisture influences the decomposition of SOM, resulting in lower SOM. But, Kerr and Ochsner [74] proved that soil moisture is one of the most influential parameters of SOM. Meanwhile, soil temperature, NPK, total N, H_2_O total N, H_2_O total organic C, and H_2_O total organic N are the most influential parameters for predicting soil organic matter. Studies show that nitrogen and total N [67], phosphorous [68], potassium [69], and NDVI [75] are influential parameters for SOM. Hence, NPK, total N, H_2_O total N, H_2_O total organic C, and H_2_O total organic N are regarded as significant predictors of the SOM in this model.

From Table 5 of fits and diagnostics for unusual observations of the regression model, we can see that the standard deviation of the residuals is 0.78. The data table also shows that the standard residual value of most observations of unusual data has a value greater than 2, resulting in a large residual, R. This indicates that these unusual observations are outliers in our dataset. By removing these outliers, the accuracy of the regression model can be increased.

**Figure 10:**
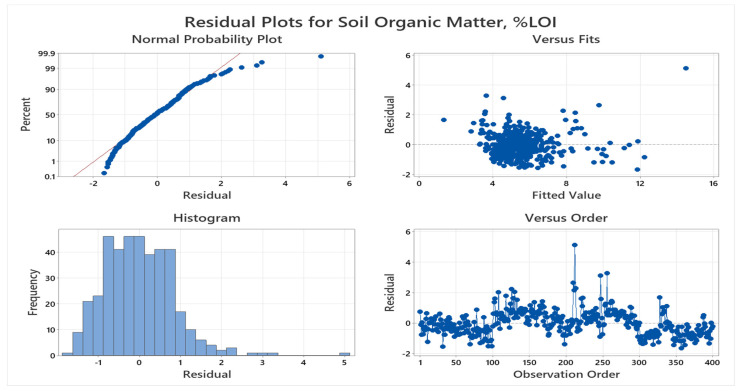
Residual plots of SOM (%).

From Figure 10, we can crosscheck the conditions for the linear model:

- Condition (1): Linearity

As the residual plot shows a completely random scatter of residual around the zero line and the Normal Probability Plot shows scatters around the diagonal line, the linear model meets the linearity condition.

- Condition (2): Nearly Normal Residuals

The histogram shows close to a bell-shaped curve. So, the linear model meets the condition of nearly normal residuals.

- Condition (3): Constant Variability

As there is no increasing or decreasing trend in the residual plot, the linear model meets the condition of constant variability.

The residual vs. observation plot shows that the data set is randomly distributed around the baseline and there is no pattern of distribution. This phenomenon indicates that the dataset of soil parameters is normally distributed.

#### 3.2.3. Machine Learning

**Figure 11:**
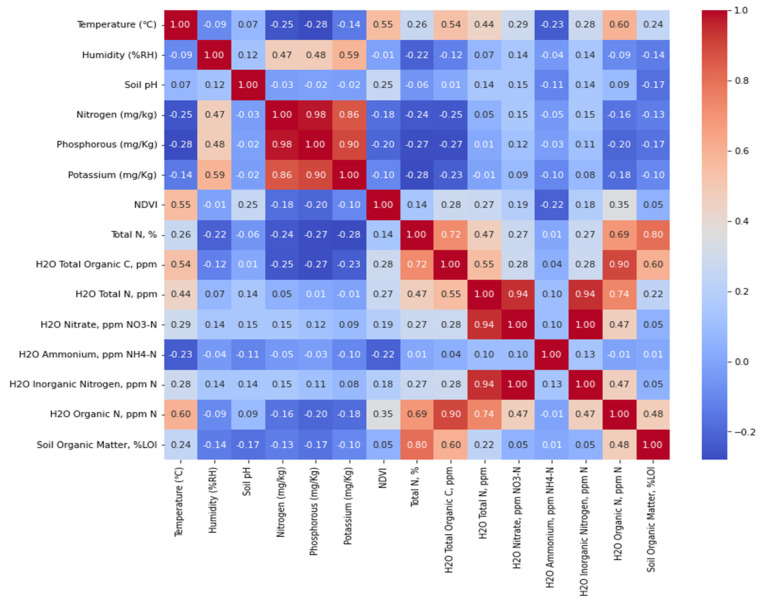
Correlation matrix of soil parameters.

The correlation matrix of the soil parameters (see Figure 11) shows that there is a strong positive connection between soil nitrogen, phosphorus, and potassium contents. These findings indicate the interdependency of soil nitrogen, phosphorus, and potassium contents and offer insights into agricultural nutrient management. The matrix also reveals a positive correlation between the total nitrogen (N) content and the total organic carbon (C) as well as the total organic nitrogen (N), suggesting that the total organic carbon and organic nitrogen in the soil are responsible for the total nitrogen content. Similarly, H_2_O total N has a strong correlation with H_2_O nitrate, H_2_O inorganic nitrogen, and H_2_O organic N. Finally, the heatmap value of soil organic matter and H_2_O ammonium is very low, which indicates that H_2_O ammonium has a very low influence on soil organic matter. Based on the correlation matrix, ‘Temperature (°C)’, ‘Humidity (%RH)’, ‘Soil pH’, ‘Phosphorous (mg/kg)’, ‘NDVI’, ‘Total N, %’, ‘H_2_O Total Organic C, ppm’, ‘H_2_O Total N, ppm’, and ‘H_2_O Organic N, ppm N’ were selected as the influential parameters for predicting soil organic matter. We used 80% of our dataset for training the model and 20% for testing and validation of the algorithm.

**Figure 12:**
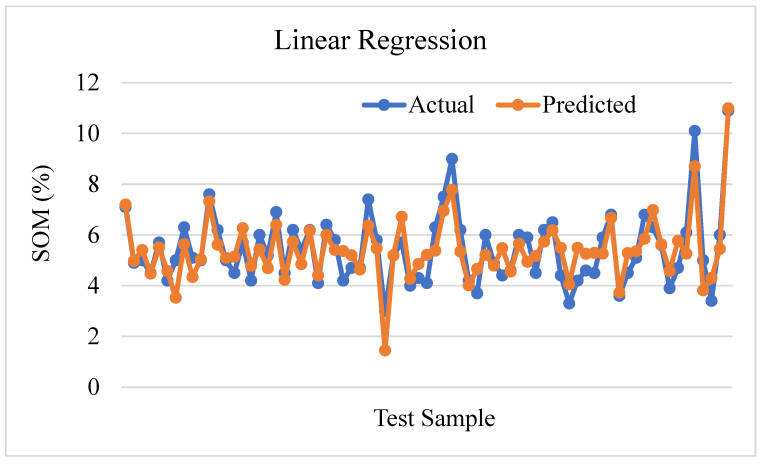
Linear regression prediction plot.

Figure 12 shows the distribution of the actual value and predicted value of soil organic matter through linear regression. The R-squared value of 0.7498 suggests that approximately 74.98% of the variability in soil organic matter can be explained by the model, indicating a reasonably suitable fit. The mean square error (MSE) value of 0.499 suggests the presence of prediction error, as the mean of the squared differences between the predicted and actual values is not equal to zero.

**Figure 13:**
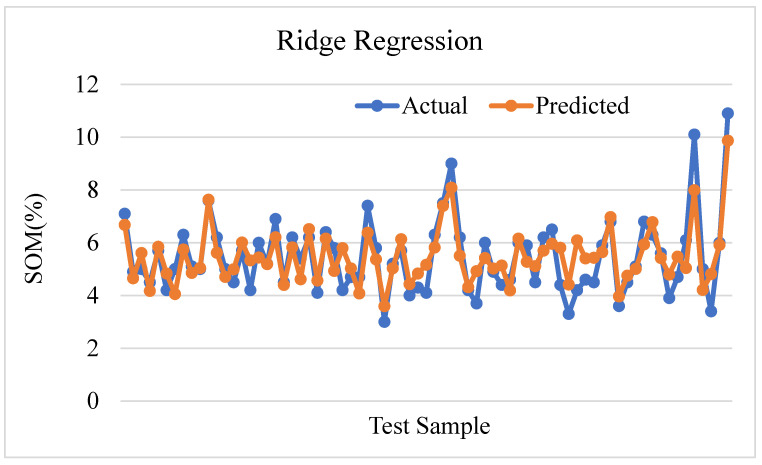
Ridge regression prediction plot.

Figure 13 displays the actual and predicted values for soil organic matter through the utilization of Ridge regression. The coefficient of determination, denoted by R-squared, has a value of 0.7304, which suggests that the Ridge regression model can account for roughly 73.04% of the variance observed in soil organic matter. This indicates a satisfactory alignment of the model with the data. The mean square error (MSE) value of 0.5386 suggests the presence of a certain degree of prediction error, given that the mean of the squared differences between the predicted and actual values is not equal to zero.

**Figure 14:**
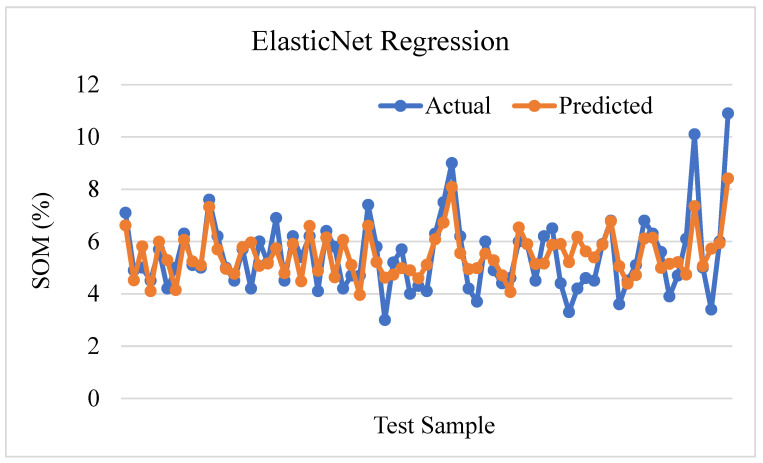
Elastic Net regression prediction plot.

Figure 14 shows a prediction plot using Elastic Net regression to estimate soil organic matter. The process of evaluation involved a comparison between the actual values and the values that were predicted. The R-squared value of 0.5387 indicated a moderate level of fit, suggesting that approximately 53.87% of the variability in soil organic matter could be explained by the model. Nevertheless, the MSE value of 0.9215 indicates notable prediction inaccuracies.

**Figure 15:**
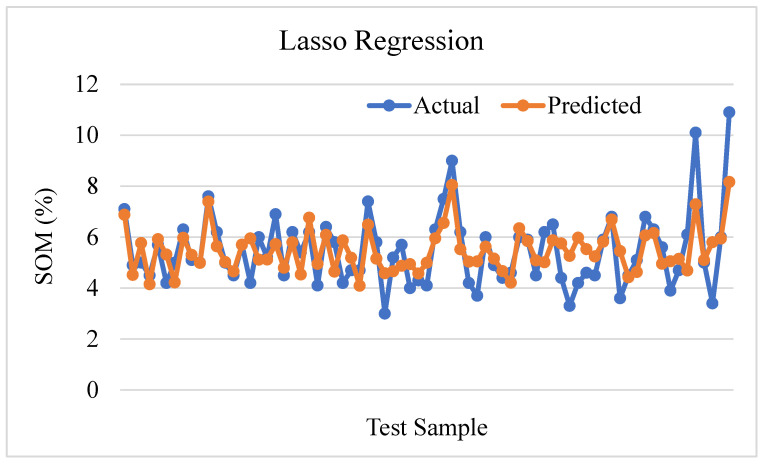
Predictive plot of Lasso regression.

The prediction plot of the Lasso regression prediction in Figure 15 shows that predicted values differ from the actual value of soil organic matter. The R-squared value of 0.5264 suggests that the Lasso regression model can explain approximately 52.64% of the variation in soil organic matter. The mean square error (MSE) of 0.9461, which is rather high, indicates a significant level of prediction error. These results imply that even while the Lasso regression model can capture certain underlying correlations and patterns, this is not a suitable fit model for predicting SOM.

**Figure 16:**
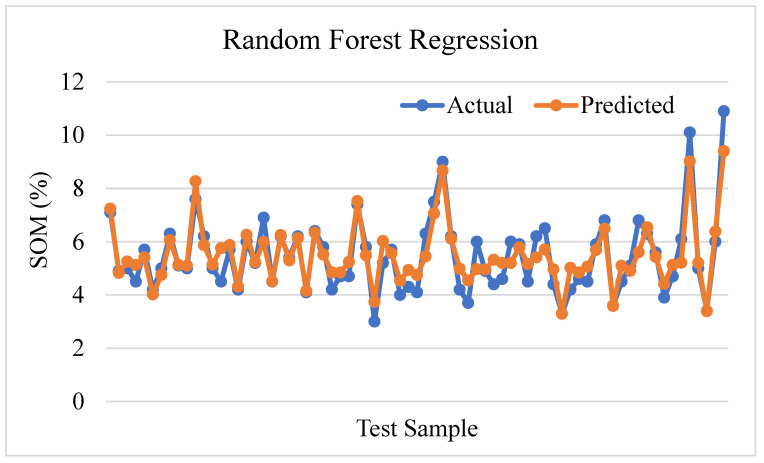
Random forest prediction plot.

The prediction plot of random forest is illustrated in Figure 16, which compares predicted values with the observed data to draw conclusions. With an R-squared of 0.8464, the random forest regression model adequately explains around 84.64% of the observed variation in soil organic matter. This demonstrates a robust connection between the soil parameters and the soil organic matter. In addition, the model’s predictions are in suitable agreement with the true values, as indicated by the small MSE of 0.3068. These findings underline the random forest regression’s promise as a trustworthy modeling tool for estimating soil organic matter.

**Figure 17:**
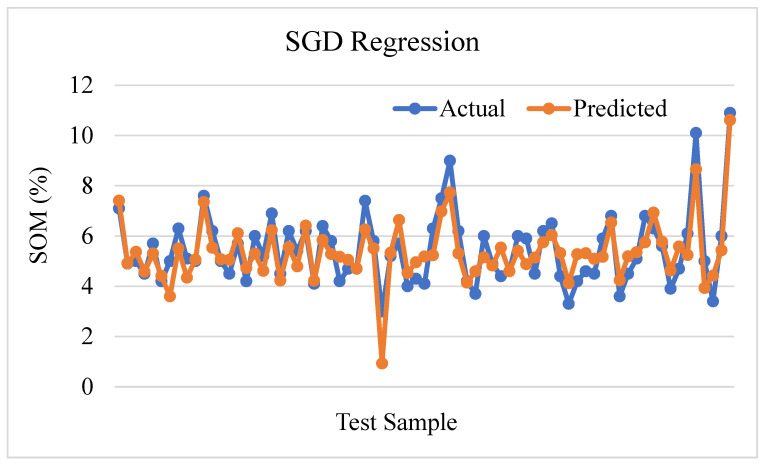
SGD regression prediction plot.

Figure 17 shows the predicted SOM with respect to test data based on the prediction of the Stochastic Gradient Descent (SGD) regression algorithm. The coefficient of determination, denoted by R-squared, of the SGD regression algorithm has a value of 0.7305, indicating that the SGD regression model explains roughly 73.05% of the variability in soil organic matter. The relationship between the independent variables and the soil organic matter appears to be moderate. The mean square error (MSE) of 0.5383 indicates a moderately high level of accuracy in predicting the soil organic matter.

**Figure 18:**
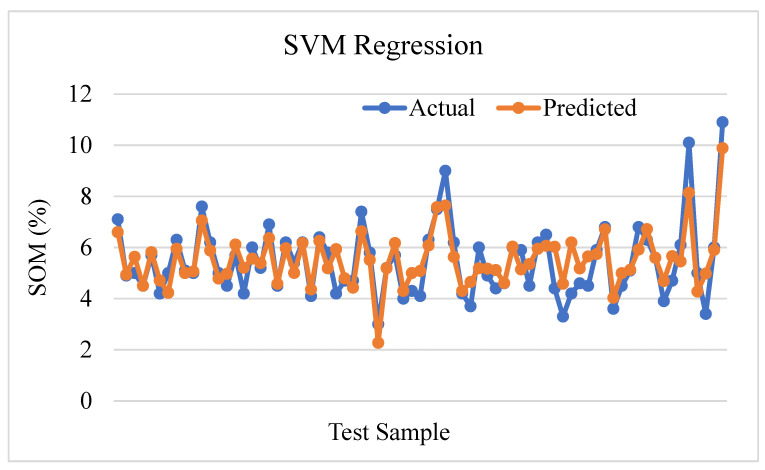
Support vector machine regression prediction plot.

Based on the analysis and prediction plot in Figure 18, the SVM regression model adequately accounts for roughly 74.004% of the observed variability in soil organic matter (R^2^ = 0.74004). An adequate correlation between the soil parameters and the soil organic matter can be explained from this. Also, the amount of prediction error is relatively low, as shown by the mean square error (MSE) of 0.5193, which is the average squared difference between the anticipated and actual values. It appears that the SVM regression technique has the potential to make precise predictions of soil organic matter.

From the above machine learning algorithms, we can see that random forest is the best algorithm for the prediction of soil organic matter. According to the R-squared value of 0.8464, the random forest regression model has the best accuracy in predicting soil organic matter. The random forest model explains approximately 84.64% of the variability of the prediction of SOM. The root mean square error (RMSE) is 0.5539, while the mean square error (MSE) is 0.3068. The average absolute difference between expected and actual values is 0.4280. Lower numbers indicate greater model fit and tighter agreement with the data. Similarly, the second-best prediction of SOM is estimated by linear regression. The accuracy of the prediction for linear regression is an R-squared value of 0.7499, RMSE value of 0.7069, and MAE value of 0.5872.

## 4. Discussions

### 4.1. Effect of Sample Size and Data Variety on SOM Prediction

The effect of sample size and data variety on SOM prediction is a critical aspect of our study. Maintaining a balance between the sufficiency and practicality of the sample pool is critical for ensuring the robustness and applicability of our SOM prediction models. Therefore, we selected three distinct land sites to reduce biased predictions and enhance model generalizability within the constraints of resource availability and logistical feasibility and collected 500 datasets to achieve a statistically sound sample size. NDVI results revealed that Montesino Ranch Farm had the highest NDVI values, as expected, due to regular soil quality management through various agricultural practices such as cow grazing, horse grazing, and the use of fertilizers. Following is Freeman Ranch, which lacked consistent soil management but had fertile soil. Star Park had the lowest NDVI among the three due to a lack of agricultural management. Selecting these different sites enabled us to increase both the size and variety of our datasets. However, this aspect can be improved by collecting more data from other sites with a greater variety of vegetation to further generalize our SOM predictive model.

### 4.2. Comparisons of Performance in SOM Prediction

Through statistical analysis of all measured parameters, we identified nine independent soil parameters in addition to the NDVI as the inputs of ML algorithms to predict SOM content. We trained seven ML algorithms using 400 datasets and used 100 additional datasets for testing the performance of the algorithms. Accuracy analysis, as presented in Table 6, revealed that random forest outperformed other ML for SOM prediction while Lasso regression and Elastic Net regression exhibited the poorest performance among others. The efficacy of random forest lies in its capability to capture complex and non-linear relationships between input features and the target variable, as well as its robustness to outliers and noisy data. Therefore, random forest emerges as a promising ML algorithm for SOM content prediction.

### 4.3. Limitations and Prospects

This study was conducted within a limited timeframe, potentially overlooking the influence of seasonal changes and land use dynamics on the SOM [76]. To mitigate this limitation, continuous data collection spanning at least a year would enable the training of more robust and generalized ML algorithms capable of considering the time series nature of the data to predict the SOM variations. Incorporating the average of data over time intervals (i.e., weekly) will allow us to train and improve our ML algorithm on a weekly basis. This approach could lead to the development of real-time predictive models capable of predicting SOM variations throughout the year, serving as a valuable tool for farmers in making informed management decisions. Therefore, future work will focus on daily monitoring of soil parameters over a year and capturing aerial imagery monthly to develop a real-time ML algorithm for SOM prediction.

## 5. Conclusions

Precision agriculture is a method of increasing the productivity, profitability, and sustainability of traditional agricultural production by gathering and analyzing data on the anticipated variability. A key element in attaining precision agriculture’s goals is the use of data collection methods. Drones and ground-based sensors are now the most effective tools for gathering plant NVDI indexes and soil characteristics and analyzing data according to design-of-experiment principles. In this study, we used a combination of soil characteristics, measured using in-ground sensors and lab-tested soil data analysis, and processed aerial images captured by drones to estimate the soil organic matter. From our initial soil data analysis, it was observed that the dataset of soil parameters is normally distributed. The regression and ANOVA analyses also show that humidity and soil pH do not have any significant correlation with SOM, but NPK, total N, H_2_O total organic carbon, and H_2_O total organic N have a strong correlation with SOM. Among all parameters, total N has the best correlation with SOM. In terms of forecasting SOM via ANOVA, our regression model accounts for 76.82% of the overall variability. The data show that the random forest algorithm provides the best accuracy in predicting soil organic matter with an accuracy of R^2^ = 0.8464, RMSE = 0.5539, and MAE value of 0.4280. The limitations of this work are the sensitivity of sensors to collect the soil parameters and the availability of farmland. The sensors we used require a certain level of soil moisture to be able to collect the soil parameters. The next phase of this research will be the automation of the data collection system using Raspberry Pi to incorporate remote data collection. Moreover, the sensors will be planted in the soil for a longer duration, and the data will be monitored continuously. This will be helpful for collecting data whenever required, and a time series dataset will be generated.

## Figures and Tables

**Table 1:** Descriptive statistics of soil parameters.

Soil Parameters	Mean	SD	Minimum	Median	Maximum
Temperature (°C)	22.81	6.13	8.30	23.40	34.50
Humidity (%RH)	26.37	12.20	5.60	23.80	72.70
Soil pH	7.63	0.92	3.00	8.03	9.00
Nitrogen (mg/kg)	34.99	15.21	4.00	29.00	94.00
Phosphorous (mg/kg)	49.35	19.70	5.00	42.50	135.00
Potassium (mg/kg)	107.14	42.86	13.00	93.50	285.00
NDVI	0.41	0.16	0.21	0.42	0.75
Total N (%)	0.29	0.10	0.14	0.27	0.88
H_2_O Total Organic C, ppm	248.71	66.54	91.90	241.35	474.90
H_2_O Total N, ppm	37.00	18.73	11.90	32.15	197.40
H_2_O Nitrate, ppm NO_3_-N	17.46	14.28	1.20	14.90	167.00
H_2_O Ammonium, ppm NH4-N	1.81	0.50	0.96	1.75	4.08
H_2_O Inorganic Nitrogen, ppm N	19.27	14.34	3.10	16.60	170.40
H_2_O Organic N, ppm N	17.75	7.09	4.30	16.50	43.90
Soil Organic Matter, %LOI	5.5622	1.748	2.9	5.3	19.6

**Table 2:** Coefficient of regression analysis.

Term	Coef	SE Coef	T-Value	*p*-Value	VIF
Constant	0.254	0.507	0.50	0.616	
Temperature (°C)	0.0471	0.0108	4.38	0.000	2.38
Humidity (%RH)	−0.00296	0.00453	−0.65	0.514	1.67
Soil pH	−0.0818	0.0502	−1.63	0.104	1.16
Nitrogen (mg/kg)	0.0771	0.0144	5.36	0.000	26.25
Phosphorous (mg/kg)	−0.0749	0.0128	−5.83	0.000	35.10
Potassium (mg/kg)	0.01457	0.00264	5.53	0.000	6.99
NDVI	−0.926	0.339	−2.74	0.006	1.58
Total N, %	16.387	0.703	23.31	0.000	2.49
H_2_O Total Organic C, ppm	0.00953	0.00169	5.65	0.000	6.91
H_2_O Total N, ppm	−0.01508	0.00375	−4.02	0.000	2.70
H_2_O Organic N, ppm N	−0.1025	0.0192	−5.33	0.000	10.18

**Table 3:** Regression model summary.

S	R-sq	R-sq (adj)	R-sq (pred)
0.853289	76.82%	76.17%	74.58%

**Table 4:** Analysis of Variance.

Source	DF	Adj SS	Adj MS	F-Value	*p*-Value
Regression	11	936.36	85.123	116.91	0.000
Temperature (°C)	1	13.95	13.952	19.16	0.000
Humidity (%RH)	1	0.31	0.311	0.43	0.514
Soil pH	1	1.93	1.934	2.66	0.104
Nitrogen (mg/kg)	1	20.91	20.907	28.71	0.000
Phosphorous (mg/kg)	1	24.73	24.726	33.96	0.000
Potassium (mg/kg)	1	22.27	22.272	30.59	0.000
NDVI	1	5.45	5.454	7.49	0.006
Total N, %	1	395.58	395.579	543.30	0.000
H_2_O Total Organic C, ppm	1	23.21	23.214	31.88	0.000
H_2_O Total N, ppm	1	11.79	11.791	16.19	0.000
H_2_O Organic N, ppm N	1	20.71	20.712	28.45	0.000
Error	388	282.50	0.728		
Total	399	1218.86			

**Table 5:** Fits and diagnostics for unusual observations.

Obs	Soil Organic Matter, %LOI	Fit	Residuals	Std Residuals		
38	5.700	5.755	−0.055	−0.07		X *
59	4.500	4.061	0.439	0.54		X
108	6.900	4.890	2.010	2.38	R *	
110	6.200	6.583	−0.383	−0.48		X
118	6.600	4.816	1.784	2.14	R	
126	5.800	3.592	2.208	2.66	R	
130	5.600	3.557	2.043	2.45	R	
210	12.400	9.771	2.629	3.13	R	
211	10.600	8.470	2.130	2.54	R	
212	19.600	14.491	5.109	6.36	R	X
213	10.100	7.829	2.271	2.69	R	
247	7.700	4.587	3.113	3.74	R	
249	5.600	5.270	0.330	0.41		X
256	6.900	3.636	3.264	3.91	R	
328	3.000	1.337	1.663	2.47	R	X

* R denotes large residual, whereas X denotes unusual observation.

**Table 6:** Prediction accuracy of different machine learning algorithms.

Model	R-Square	Mean Square Error	Root Mean Square Error	Mean Absolute Error
Linear Regression	0.7499	0.4997	0.7069	0.5872
Elastic Net Regression	0.5387	0.9215	0.9599	0.7398
Lasso Regression	0.5264	0.9462	0.9727	0.7423
Ridge Regression	0.7304	0.5386	0.7339	0.5949
Random Forest	**0.8464**	**0.3068**	**0.5539**	**0.4280**
SGD Regression	0.7301	0.5392	0.7343	0.6198
SVM Regression	0.7400	0.5193	0.7206	0.5430

## Data Availability

Data supporting reported results will be provided upon request to the corresponding authors.
